# Supplementary material for: Protein-Mediated Electroporation in a Cardiac Voltage-Sensing Domain Due to an nsPEF Stimulus
Source: Int J Mol Sci. 2023 Jul 13;24(14):11397. doi: 10.3390/ijms241411397 (PMC10379607; doi:10.3390/ijms241411397)
Supplement: Supplementary file 1 [file ijms-24-11397-s001.zip › ijms-2399289-supplementary.pdf]

Supplementary Information

1 membranes rotate 90°

Representation of different POPC rearrangement at different external $\vec{E}_z$ (from 0.14 V/nm to 0.2 V/nm) after 50 ns of simulation. In all examples can been observed a turn of the POPC bilayer in 90°. Figure S1

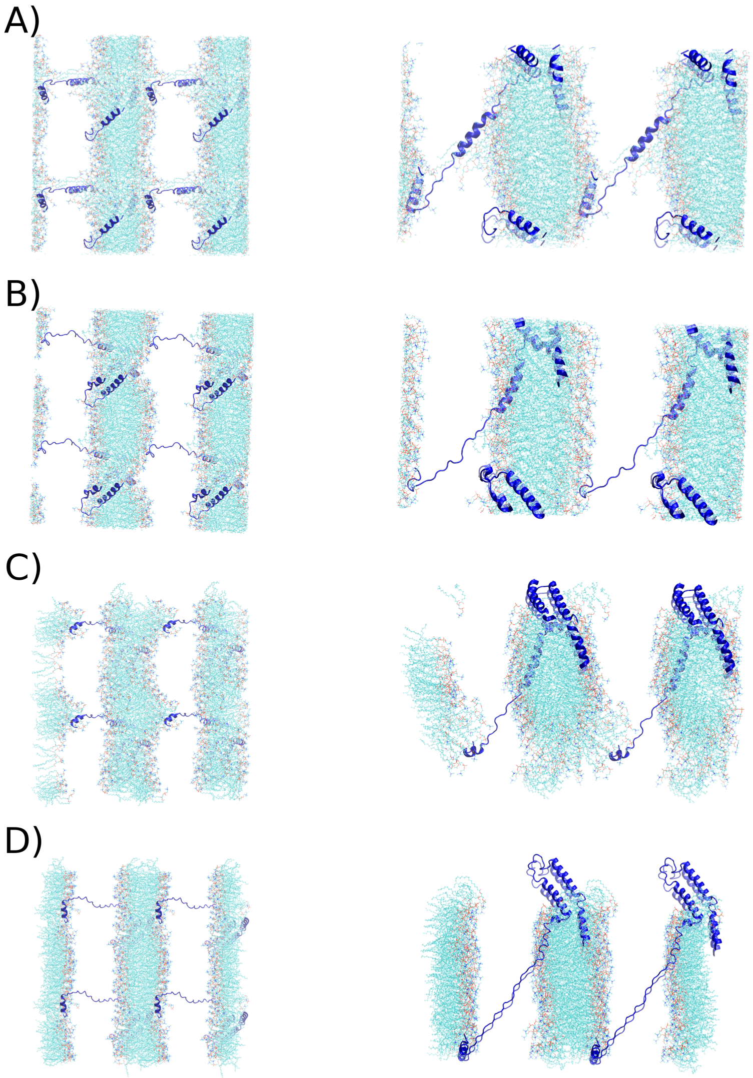

Figure S1: Examples of representation in lines of the POPC bileyar after 50 ns of simulation. All images in left are view above x-y plane, and in the right are view above z-x plane. A) Example of POPC rearrangement af $\vec{E}_z$=0.14 V/nm. B) Example of POPC rearrangement af $\vec{E}_z$=0.16 V/nm. C) Example of POPC rearrangement af $\vec{E}_z$=0.18 V/nm. D) Example of POPC rearrangement af $\vec{E}_z$=0.2 V/nm.

2 RMSD of the selected replicas after the first filter and after the second filter

The Figure S2 are the RMSD as a function of time of the replicas that afford the condition of the first filter (derivative of the RMSD minor than 15% at the last 10 ns) and the replicas that pass the second filter (Have a RMSD final minor to 0.35 V/nm), given a total of 142 replicas.

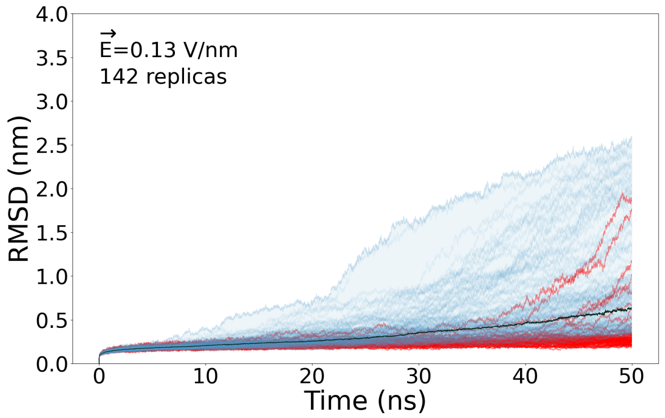

Figure S2: RMSDs of the 142 replicas that pass the first and second filter. In red the replicas that not pass the filters.

3 Examples of VSD rearrangement whit water crossing de bilayer at different ending VSD RMSD.

The next Figure shows four examples at different final RMSDs of the VSD structure together with the water inside the VSDs, representation extracted from the 200 replicas under an external $\vec{E}_z$=0.13 V/nm. This gives an idea of the structure of the VSD as a function of the RMSD.

4 3D cube representing the Tuples covering the phase space

The next Figure represent all tuples, to be sure that the data is covering the phase space to be able to calculate ΔG^0 between clusters.

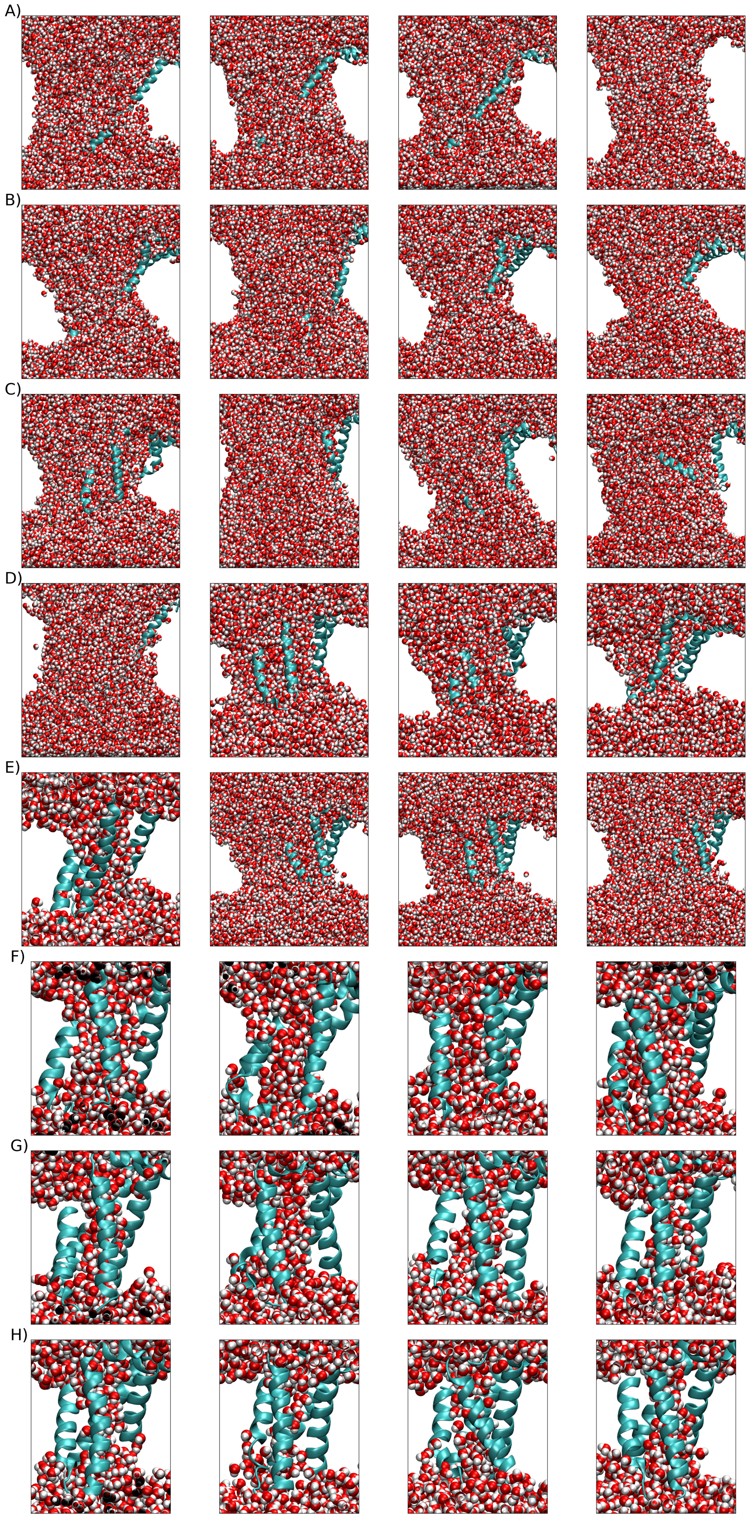
A)
B)
C)
D)
E)
F)
G)
H)

Figure 3: In all images the protein is represented in sky blue cartoon, and water in van der waals representation. A) Images of the four replicas ending in the higher RMSD. B) Images of four replicas with the RMSD more close to RMSD=2 nm below it. C) Images of four replicas with the RMSD more close to RMSD=1.8 nm below it. D) Images of four replicas with the RMSD more close to RMSD=1.6 nm below it. E) Images of four replicas with the RMSD more close to RMSD=1.4 nm below it. E) Images of four replicas with the RMSD more close to RMSD=1.2 nm below it. E) Images of four replicas with the RMSD more close to RMSD=0.8 nm below it. E) Images of four replicas with the RMSD more close to RMSD=0.4 nm below it. E) Images of four replicas with the RMSD more close to RMSD=0.3 nm below it

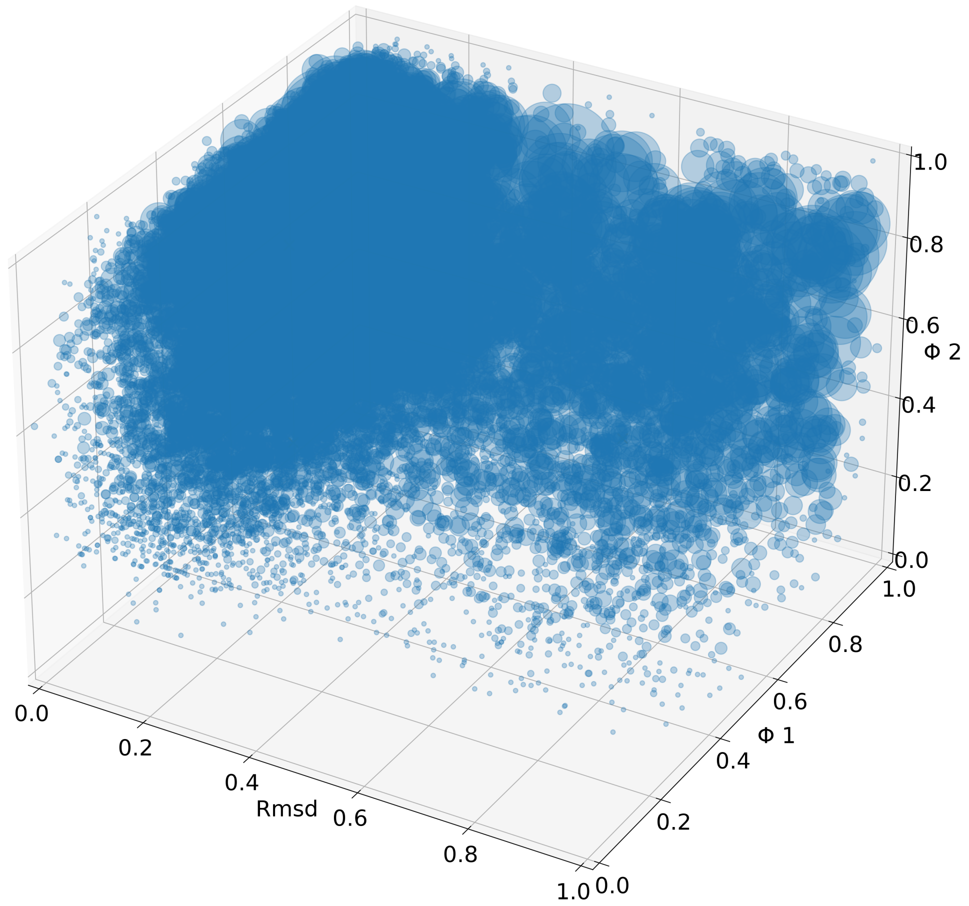

Figure S4: 3D cube with all Tuples. ϕ=FNC.

5 Minimum distance beteewen VSDs and it periodic image

In Figure S5 can be observe that none of the 200 replicas at $\vec{E}_z$=0.13 V/nm simulation see it periodic images (cut-off = 1.2 nm).

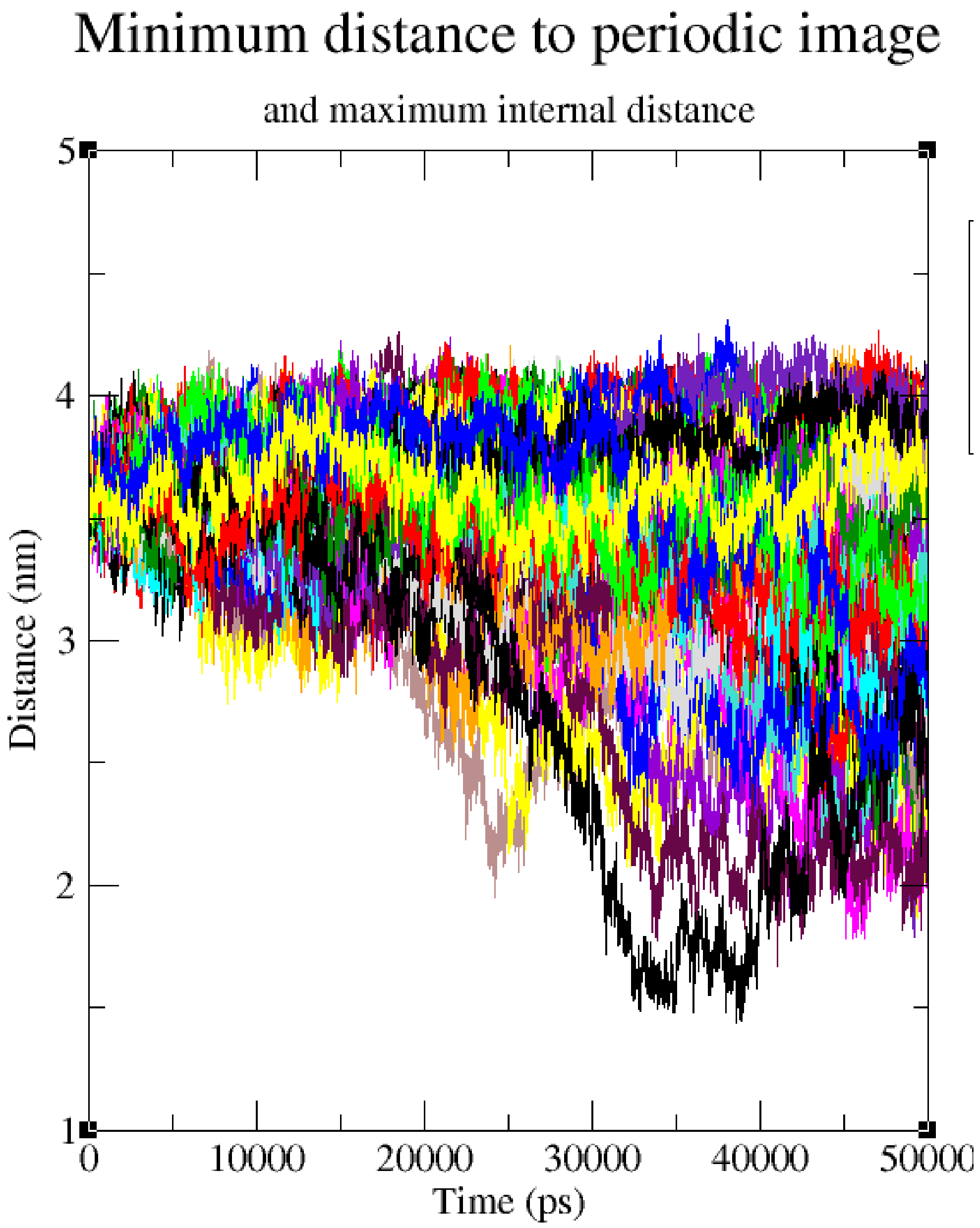

Figure S5: Minimum distance beteewen VSDs and it periodic imag of the 200 replicas at $\vec{E}_z$=0.13 V/nm.
